# Elucidating the role of Bovine Leukemia Virus encoded micro-RNAs

**DOI:** 10.1186/1742-4690-11-S1-O62

**Published:** 2014-01-07

**Authors:** Keith Durkin, Nicolas Rosewick, Mélanie Momont, Wannes Thys, Arsène Burny, Michel Georges, Anne Van den Broeke

**Affiliations:** 1 Unit of Animal Genomics, Groupe Interdisciplinaire Génoprotéomique Appliquée, Université de Liège, Liège, Belgium; 2 Laboratory of Experimental Hematology, Institut Jules Bordet, Université Libre de Bruxelles, Brussels, Belgium

## 

Bovine Leukemia Virus (BLV) and its close relative Human T-cell leukemia virus-1 (HTLV-1) display similar patterns of pathogenesis and genome organisation. The natural host of BLV is cattle, however it is possible to experimentally infect sheep with the virus. Infected sheep develop tumors following a significantly reduced latency period compared to cattle (20 months on average), making for an attractive cancer model. Like HTLV-1, BLV mRNAs/proteins transcribed from the 5’ LTR are silenced in tumors. However, our group and others have recently reported the presence of five highly expressed micro-RNAs transcribed from the BLV genome via a noncanonical RNA polymerase III pathway in BLV induced tumors. It has been noted that one of these micro-RNAs (BLV-miR-B4-3p) shares a seed sequence with miR-29, a regulator of the tumor suppressor HBP1. This observation points to one potential role for a single micro-RNA, however the role of the remaining micro-RNAs remains to be uncovered. In order to further explore potential targets of the BLV micro-RNAs we have carried out high throughput RNA sequencing of a number of experientially induced ovine and natural bovine BLV tumors, in addition to ovine derived BLV tumor cell lines. As a result we have identified a target of the viral-microRNAs and have begun exploring its role in the life cycle of the virus and its potential contribution to tumorigenesis. Data describing the results obtained to date will be discussed.

